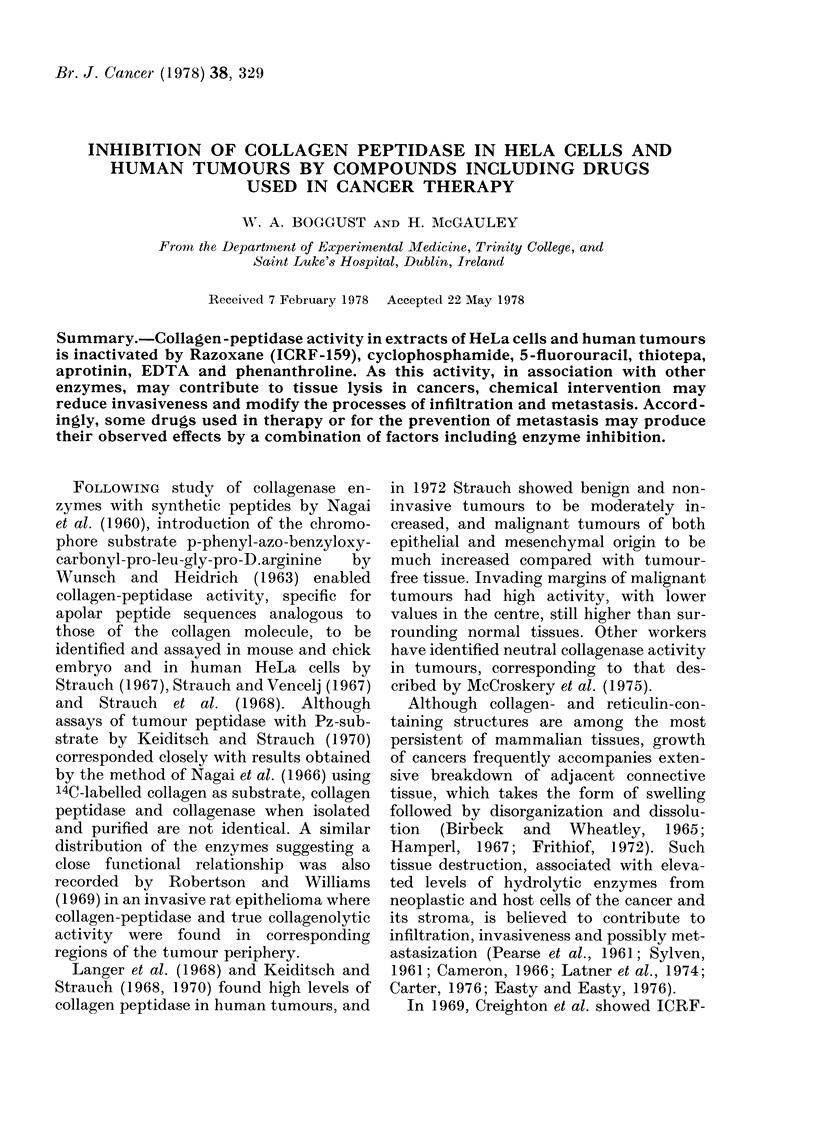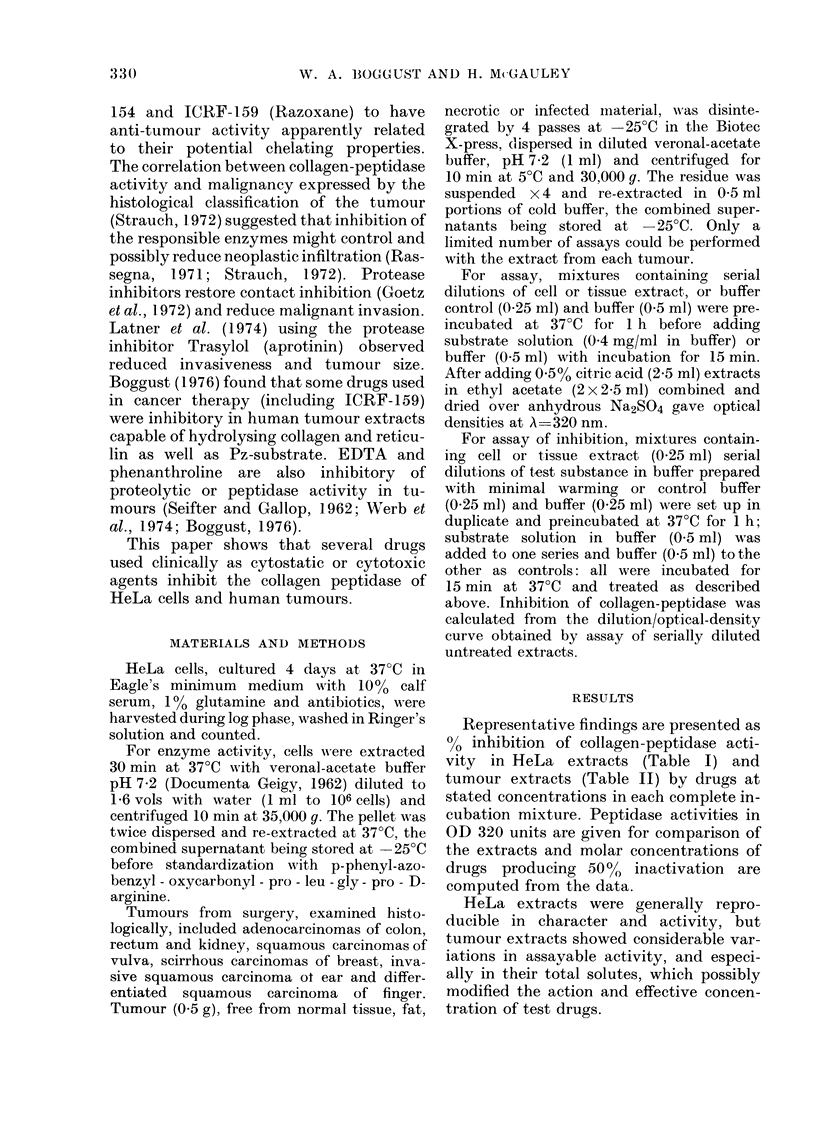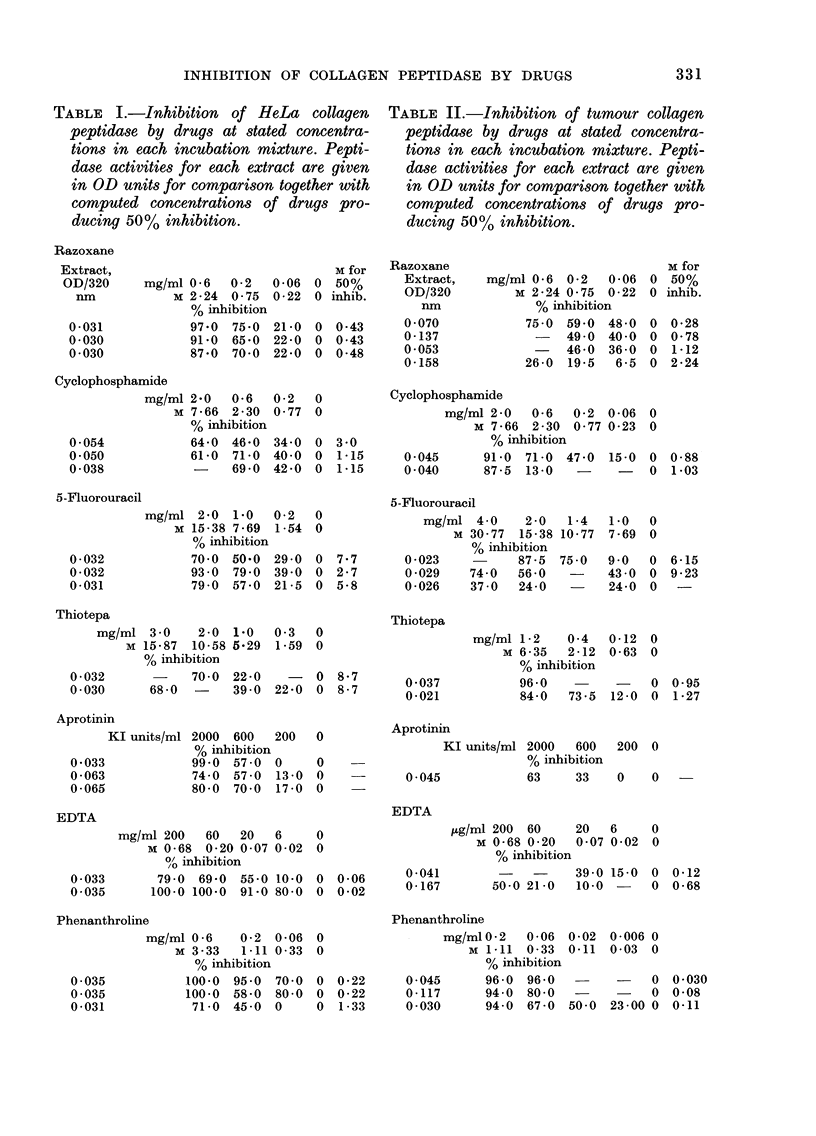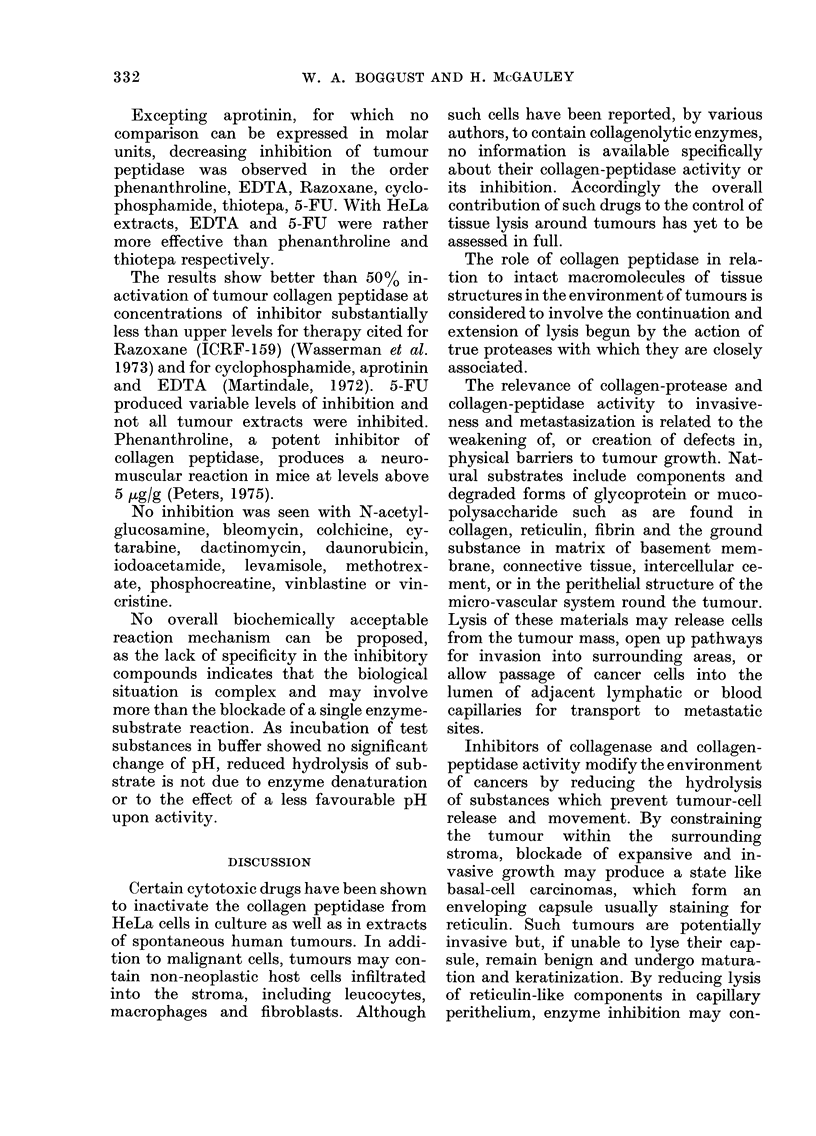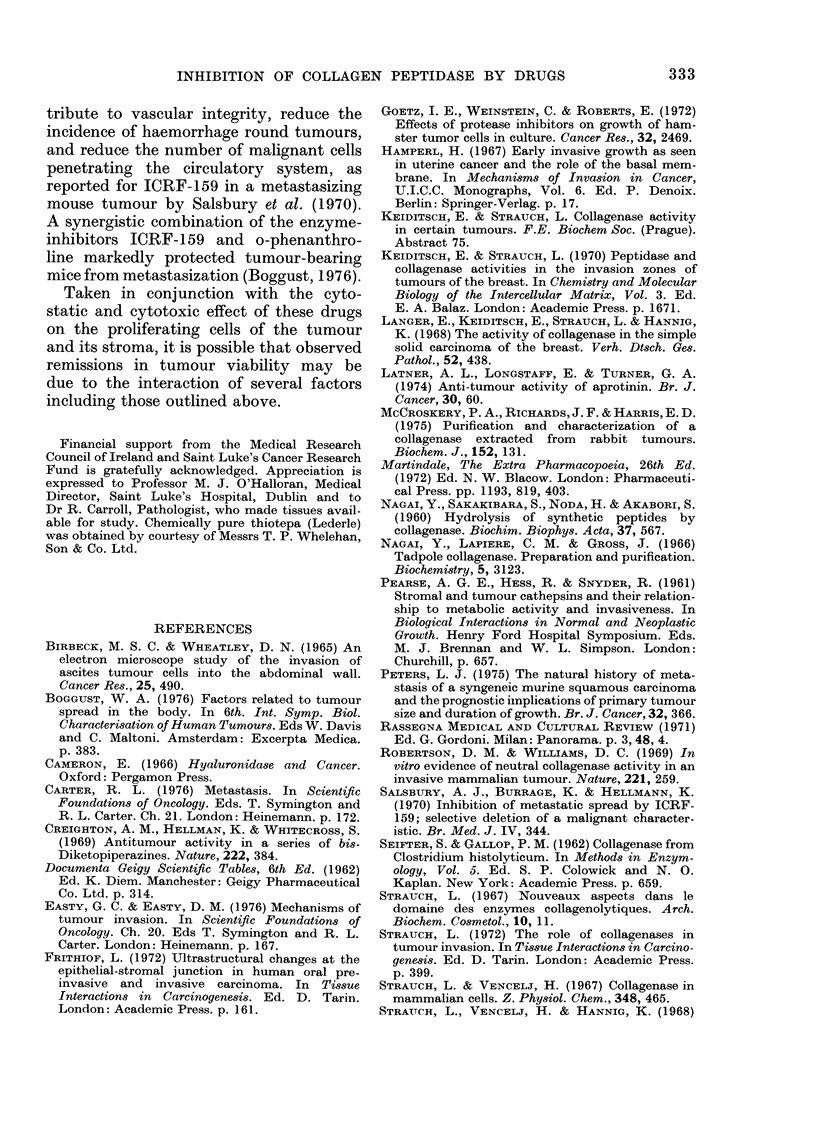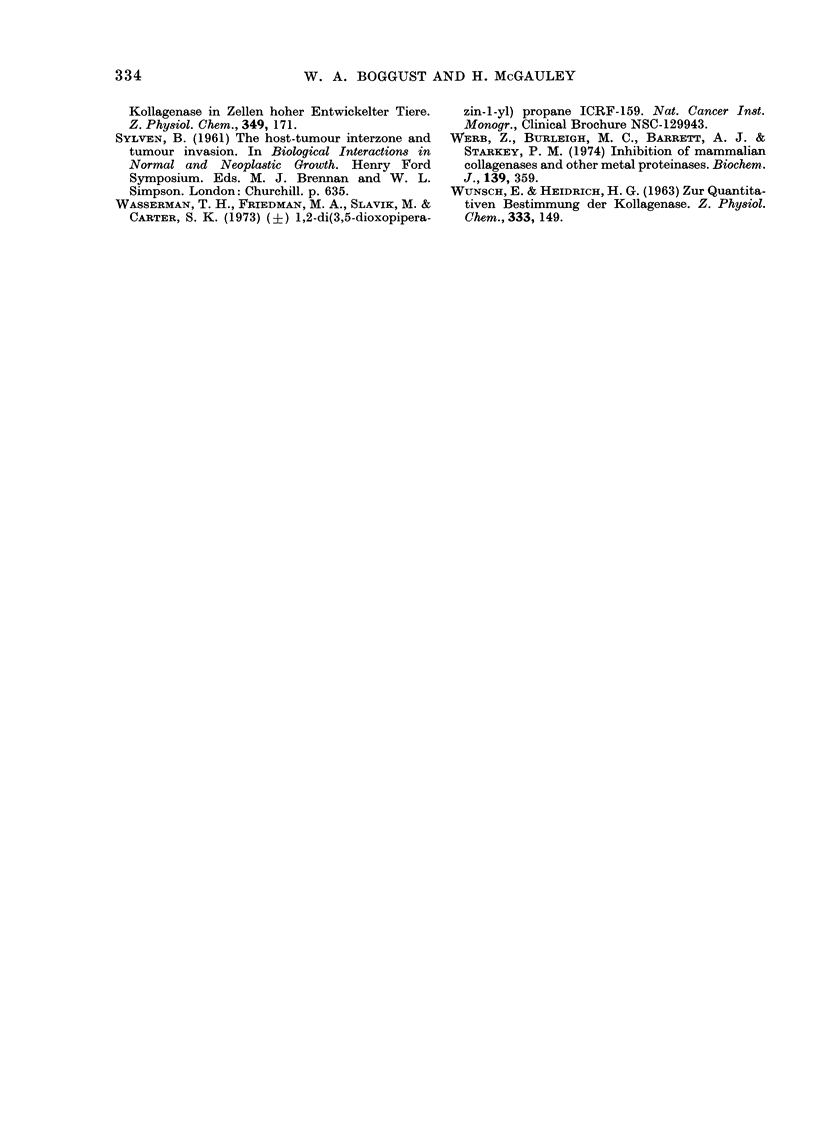# Inhibition of collagen peptidase in HeLa cells and human tumours by compounds including drugs used in cancer therapy.

**DOI:** 10.1038/bjc.1978.206

**Published:** 1978-08

**Authors:** W. A. Boggust, H. McGauley

## Abstract

Collagen-peptidase activity in extracts of HeLa cells and human tumours is inactivated by Razoxane (ICRF-159), cyclophosphamide, 5-fluorouracil, thiotepa, aprotinin, EDTA and phenanthroline. As this activity, in association with other enzymes, may contribute to tissue lysis in cancers, chemical intervention may reduce invasiveness and modify the processes of infiltration and metastasis. Accordingly, some drugs used in therapy or for the prevention of metastasis may produce their observed effects by a combination of factors including enzyme inhibition.


*Br. J. Cancer* (1978) **38**, 329

### INHIBITION OF COLLAGEN PEPTIDASE IN HELA CELLS AND HUMAN TUMOURS BY COMPOUNDS INCLUDING DRUGS USED IN CANCER THERAPY

W. A. BOGGUST AND H. McGAULEY

*From the Department of Experimental Medicine, Trinity College, and Saint Luke's Hospital, Dublin, Ireland*

Received 7 February 1978 Accepted 22 May 1978

**Summary.—Collagen-peptidase activity in extracts of HeLa cells and human tumours is inactivated by Razoxane (ICRF-159), cyclophosphamide, 5-fluorouracil, thiotepa, aprotinin, EDTA and phenanthroline. As this activity, in association with other enzymes, may contribute to tissue lysis in cancers, chemical intervention may reduce invasiveness and modify the processes of infiltration and metastasis. Accordingly, some drugs used in therapy or for the prevention of metastasis may produce their observed effects by a combination of factors including enzyme inhibition.**

FOLLOWING study of collagenase enzymes with synthetic peptides by Nagai *et al.* (1960), introduction of the chromophore substrate p-phenyl-azo-benzyloxy-carbonyl-pro-leu-gly-pro-D.arginine by Wunsch and Heidrich (1963) enabled collagen-peptidase activity, specific for apolar peptide sequences analogous to those of the collagen molecule, to be identified and assayed in mouse and chick embryo and in human HeLa cells by Strauch (1967), Strauch and Vencelj (1967) and Strauch *et al.* (1968). Although assays of tumour peptidase with Pz-substrate by Keiditsch and Strauch (1970) corresponded closely with results obtained by the method of Nagai *et al.* (1966) using $^{14}C$-labelled collagen as substrate, collagen peptidase and collagenase when isolated and purified are not identical. A similar distribution of the enzymes suggesting a close functional relationship was also recorded by Robertson and Williams (1969) in an invasive rat epithelioma where collagen-peptidase and true collagenolytic activity were found in corresponding regions of the tumour periphery.

Langer *et al.* (1968) and Keiditsch and Strauch (1968, 1970) found high levels of collagen peptidase in human tumours, and in 1972 Strauch showed benign and non-invasive tumours to be moderately increased, and malignant tumours of both epithelial and mesenchymal origin to be much increased compared with tumour-free tissue. Invading margins of malignant tumours had high activity, with lower values in the centre, still higher than surrounding normal tissues. Other workers have identified neutral collagenase activity in tumours, corresponding to that described by McCroskery *et al.* (1975).

Although collagen- and reticulin-containing structures are among the most persistent of mammalian tissues, growth of cancers frequently accompanies extensive breakdown of adjacent connective tissue, which takes the form of swelling followed by disorganization and dissolution (Birbeck and Wheatley, 1965; Hamperl, 1967; Frithiof, 1972). Such tissue destruction, associated with elevated levels of hydrolytic enzymes from neoplastic and host cells of the cancer and its stroma, is believed to contribute to infiltration, invasiveness and possibly metastasization (Pearse *et al.*, 1961; Sylven, 1961; Cameron, 1966; Latner *et al.*, 1974; Carter, 1976; Easty and Easty, 1976).

In 1969, Creighton *et al.* showed ICRF-

154 and ICRF-159 (Razoxane) to have anti-tumour activity apparently related to their potential chelating properties. The correlation between collagen-peptidase activity and malignancy expressed by the histological classification of the tumour (Strauch, 1972) suggested that inhibition of the responsible enzymes might control and possibly reduce neoplastic infiltration (Rassegna, 1971; Strauch, 1972). Protease inhibitors restore contact inhibition (Goetz *et al.*, 1972) and reduce malignant invasion. Latner *et al.* (1974) using the protease inhibitor Trasylol (aprotinin) observed reduced invasiveness and tumour size. Boggust (1976) found that some drugs used in cancer therapy (including ICRF-159) were inhibitory in human tumour extracts capable of hydrolysing collagen and reticulin as well as Pz-substrate. EDTA and phenanthroline are also inhibitory of proteolytic or peptidase activity in tumours (Seifter and Gallop, 1962; Werb *et al.*, 1974; Boggust, 1976).

This paper shows that several drugs used clinically as cytostatic or cytotoxic agents inhibit the collagen peptidase of HeLa cells and human tumours.

### MATERIALS AND METHODS

HeLa cells, cultured 4 days at 37°C in Eagle's minimum medium with 10% calf serum, 1% glutamine and antibiotics, were harvested during log phase, washed in Ringer's solution and counted.

For enzyme activity, cells were extracted 30 min at 37°C with veronal-acetate buffer pH 7·2 (Documenta Geigy, 1962) diluted to 1·6 vols with water (1 ml to $10^6$ cells) and centrifuged 10 min at 35,000 *g*. The pellet was twice dispersed and re-extracted at 37°C, the combined supernatant being stored at −25°C before standardization with p-phenyl-azo-benzyl - oxycarbonyl - pro - leu - gly - pro - D-arginine.

Tumours from surgery, examined histologically, included adenocarcinomas of colon, rectum and kidney, squamous carcinomas of vulva, scirrhous carcinomas of breast, invasive squamous carcinoma of ear and differentiated squamous carcinoma of finger. Tumour (0·5 g), free from normal tissue, fat, necrotic or infected material, was disintegrated by 4 passes at −25°C in the Biotec X-press, dispersed in diluted veronal-acetate buffer, pH 7·2 (1 ml) and centrifuged for 10 min at 5°C and 30,000 *g*. The residue was suspended ×4 and re-extracted in 0·5 ml portions of cold buffer, the combined supernatants being stored at −25°C. Only a limited number of assays could be performed with the extract from each tumour.

For assay, mixtures containing serial dilutions of cell or tissue extract, or buffer control (0·25 ml) and buffer (0·5 ml) were preincubated at 37°C for 1 h before adding substrate solution (0·4 mg/ml in buffer) or buffer (0·5 ml) with incubation for 15 min. After adding 0·5% citric acid (2·5 ml) extracts in ethyl acetate (2×2·5 ml) combined and dried over anhydrous $Na_2SO_4$ gave optical densities at $\lambda$=320 nm.

For assay of inhibition, mixtures containing cell or tissue extract (0·25 ml) serial dilutions of test substance in buffer prepared with minimal warming or control buffer (0·25 ml) and buffer (0·25 ml) were set up in duplicate and preincubated at 37°C for 1 h; substrate solution in buffer (0·5 ml) was added to one series and buffer (0·5 ml) to the other as controls: all were incubated for 15 min at 37°C and treated as described above. Inhibition of collagen-peptidase was calculated from the dilution/optical-density curve obtained by assay of serially diluted untreated extracts.

### RESULTS

Representative findings are presented as % inhibition of collagen-peptidase activity in HeLa extracts (Table I) and tumour extracts (Table II) by drugs at stated concentrations in each complete incubation mixture. Peptidase activities in OD 320 units are given for comparison of the extracts and molar concentrations of drugs producing 50% inactivation are computed from the data.

HeLa extracts were generally reproducible in character and activity, but tumour extracts showed considerable variations in assayable activity, and especially in their total solutes, which possibly modified the action and effective concentration of test drugs.

TABLE I.—*Inhibition of HeLa collagen peptidase by drugs at stated concentrations in each incubation mixture. Peptidase activities for each extract are given in OD units for comparison together with computed concentrations of drugs producing 50% inhibition.*

Razoxane

| Extract, OD/320 nm | | | | | | M for 50% inhib. |
|---|---|---|---|---|---|---|
| | mg/ml | 0·6 | 0·2 | 0·06 | 0 | |
| | M | 2·24 | 0·75 | 0·22 | 0 | |
| | | % inhibition | | | | |
| 0·031 | | 97·0 | 75·0 | 21·0 | 0 | 0·43 |
| 0·030 | | 91·0 | 65·0 | 22·0 | 0 | 0·43 |
| 0·030 | | 87·0 | 70·0 | 22·0 | 0 | 0·48 |

Cyclophosphamide

| | | | | | | |
|---|---|---|---|---|---|---|
| | mg/ml | 2·0 | 0·6 | 0·2 | 0 | |
| | M | 7·66 | 2·30 | 0·77 | 0 | |
| | | % inhibition | | | | |
| 0·054 | | 64·0 | 46·0 | 34·0 | 0 | 3·0 |
| 0·050 | | 61·0 | 71·0 | 40·0 | 0 | 1·15 |
| 0·038 | | — | 69·0 | 42·0 | 0 | 1·15 |

5-Fluorouracil

| | | | | | | |
|---|---|---|---|---|---|---|
| | mg/ml | 2·0 | 1·0 | 0·2 | 0 | |
| | M | 15·38 | 7·69 | 1·54 | 0 | |
| | | % inhibition | | | | |
| 0·032 | | 70·0 | 50·0 | 29·0 | 0 | 7·7 |
| 0·032 | | 93·0 | 79·0 | 39·0 | 0 | 2·7 |
| 0·031 | | 79·0 | 57·0 | 21·5 | 0 | 5·8 |

Thiotepa

| | | | | | | | |
|---|---|---|---|---|---|---|---|
| | mg/ml | 3·0 | 2·0 | 1·0 | 0·3 | 0 | |
| | M | 15·87 | 10·58 | 5·29 | 1·59 | 0 | |
| | | % inhibition | | | | | |
| 0·032 | | — | 70·0 | 22·0 | — | 0 | 8·7 |
| 0·030 | | 68·0 | — | 39·0 | 22·0 | 0 | 8·7 |

Aprotinin

| | | | | | | |
|---|---|---|---|---|---|---|
| | KI units/ml | 2000 | 600 | 200 | 0 | |
| | | % inhibition | | | | |
| 0·033 | | 99·0 | 57·0 | 0 | 0 | — |
| 0·063 | | 74·0 | 57·0 | 13·0 | 0 | — |
| 0·065 | | 80·0 | 70·0 | 17·0 | 0 | — |

EDTA

| | | | | | | | |
|---|---|---|---|---|---|---|---|
| | mg/ml | 200 | 60 | 20 | 6 | 0 | |
| | M | 0·68 | 0·20 | 0·07 | 0·02 | 0 | |
| | | % inhibition | | | | | |
| 0·033 | | 79·0 | 69·0 | 55·0 | 10·0 | 0 | 0·06 |
| 0·035 | | 100·0 | 100·0 | 91·0 | 80·0 | 0 | 0·02 |

Phenanthroline

| | | | | | | |
|---|---|---|---|---|---|---|
| | mg/ml | 0·6 | 0·2 | 0·06 | 0 | |
| | M | 3·33 | 1·11 | 0·33 | 0 | |
| | | % inhibition | | | | |
| 0·035 | | 100·0 | 95·0 | 70·0 | 0 | 0·22 |
| 0·035 | | 100·0 | 58·0 | 80·0 | 0 | 0·22 |
| 0·031 | | 71·0 | 45·0 | 0 | 0 | 1·33 |

TABLE II.—*Inhibition of tumour collagen peptidase by drugs at stated concentrations in each incubation mixture. Peptidase activities for each extract are given in OD units for comparison together with computed concentrations of drugs producing 50% inhibition.*

Razoxane

| Extract, OD/320 nm | | | | | | M for 50% inhib. |
|---|---|---|---|---|---|---|
| | mg/ml | 0·6 | 0·2 | 0·06 | 0 | |
| | M | 2·24 | 0·75 | 0·22 | 0 | |
| | | % inhibition | | | | |
| 0·070 | | 75·0 | 59·0 | 48·0 | 0 | 0·28 |
| 0·137 | | — | 49·0 | 40·0 | 0 | 0·78 |
| 0·053 | | — | 46·0 | 36·0 | 0 | 1·12 |
| 0·158 | | 26·0 | 19·5 | 6·5 | 0 | 2·24 |

Cyclophosphamide

| | | | | | | | |
|---|---|---|---|---|---|---|---|
| | mg/ml | 2·0 | 0·6 | 0·2 | 0·06 | 0 | |
| | M | 7·66 | 2·30 | 0·77 | 0·23 | 0 | |
| | | % inhibition | | | | | |
| 0·045 | | 91·0 | 71·0 | 47·0 | 15·0 | 0 | 0·88 |
| 0·040 | | 87·5 | 13·0 | — | — | 0 | 1·03 |

5-Fluorouracil

| | | | | | | | |
|---|---|---|---|---|---|---|---|
| | mg/ml | 4·0 | 2·0 | 1·4 | 1·0 | 0 | |
| | M | 30·77 | 15·38 | 10·77 | 7·69 | 0 | |
| | | % inhibition | | | | | |
| 0·023 | | — | 87·5 | 75·0 | 9·0 | 0 | 6·15 |
| 0·029 | | 74·0 | 56·0 | — | 43·0 | 0 | 9·23 |
| 0·026 | | 37·0 | 24·0 | — | 24·0 | 0 | — |

Thiotepa

| | | | | | | |
|---|---|---|---|---|---|---|
| | mg/ml | 1·2 | 0·4 | 0·12 | 0 | |
| | M | 6·35 | 2·12 | 0·63 | 0 | |
| | | % inhibition | | | | |
| 0·037 | | 96·0 | — | — | 0 | 0·95 |
| 0·021 | | 84·0 | 73·5 | 12·0 | 0 | 1·27 |

Aprotinin

| | | | | | | |
|---|---|---|---|---|---|---|
| | KI units/ml | 2000 | 600 | 200 | 0 | |
| | | % inhibition | | | | |
| 0·045 | | 63 | 33 | 0 | 0 | — |

EDTA

| | | | | | | | |
|---|---|---|---|---|---|---|---|
| | µg/ml | 200 | 60 | 20 | 6 | 0 | |
| | M | 0·68 | 0·20 | 0·07 | 0·02 | 0 | |
| | | % inhibition | | | | | |
| 0·041 | | — | — | 39·0 | 15·0 | 0 | 0·12 |
| 0·167 | | 50·0 | 21·0 | 10·0 | — | 0 | 0·68 |

Phenanthroline

| | | | | | | | |
|---|---|---|---|---|---|---|---|
| | mg/ml | 0·2 | 0·06 | 0·02 | 0·006 | 0 | |
| | M | 1·11 | 0·33 | 0·11 | 0·03 | 0 | |
| | | % inhibition | | | | | |
| 0·045 | | 96·0 | 96·0 | — | — | 0 | 0·030 |
| 0·117 | | 94·0 | 80·0 | — | — | 0 | 0·08 |
| 0·030 | | 94·0 | 67·0 | 50·0 | 23·00 | 0 | 0·11 |

Excepting aprotinin, for which no comparison can be expressed in molar units, decreasing inhibition of tumour peptidase was observed in the order phenanthroline, EDTA, Razoxane, cyclophosphamide, thiotepa, 5-FU. With HeLa extracts, EDTA and 5-FU were rather more effective than phenanthroline and thiotepa respectively.

The results show better than 50% inactivation of tumour collagen peptidase at concentrations of inhibitor substantially less than upper levels for therapy cited for Razoxane (ICRF-159) (Wasserman *et al.* 1973) and for cyclophosphamide, aprotinin and EDTA (Martindale, 1972). 5-FU produced variable levels of inhibition and not all tumour extracts were inhibited. Phenanthroline, a potent inhibitor of collagen peptidase, produces a neuromuscular reaction in mice at levels above 5 $\mu$g/g (Peters, 1975).

No inhibition was seen with N-acetylglucosamine, bleomycin, colchicine, cytarabine, dactinomycin, daunorubicin, iodoacetamide, levamisole, methotrexate, phosphocreatine, vinblastine or vincristine.

No overall biochemically acceptable reaction mechanism can be proposed, as the lack of specificity in the inhibitory compounds indicates that the biological situation is complex and may involve more than the blockade of a single enzyme-substrate reaction. As incubation of test substances in buffer showed no significant change of pH, reduced hydrolysis of substrate is not due to enzyme denaturation or to the effect of a less favourable pH upon activity.

### DISCUSSION

Certain cytotoxic drugs have been shown to inactivate the collagen peptidase from HeLa cells in culture as well as in extracts of spontaneous human tumours. In addition to malignant cells, tumours may contain non-neoplastic host cells infiltrated into the stroma, including leucocytes, macrophages and fibroblasts. Although such cells have been reported, by various authors, to contain collagenolytic enzymes, no information is available specifically about their collagen-peptidase activity or its inhibition. Accordingly the overall contribution of such drugs to the control of tissue lysis around tumours has yet to be assessed in full.

The role of collagen peptidase in relation to intact macromolecules of tissue structures in the environment of tumours is considered to involve the continuation and extension of lysis begun by the action of true proteases with which they are closely associated.

The relevance of collagen-protease and collagen-peptidase activity to invasiveness and metastasization is related to the weakening of, or creation of defects in, physical barriers to tumour growth. Natural substrates include components and degraded forms of glycoprotein or mucopolysaccharide such as are found in collagen, reticulin, fibrin and the ground substance in matrix of basement membrane, connective tissue, intercellular cement, or in the perithelial structure of the micro-vascular system round the tumour. Lysis of these materials may release cells from the tumour mass, open up pathways for invasion into surrounding areas, or allow passage of cancer cells into the lumen of adjacent lymphatic or blood capillaries for transport to metastatic sites.

Inhibitors of collagenase and collagen-peptidase activity modify the environment of cancers by reducing the hydrolysis of substances which prevent tumour-cell release and movement. By constraining the tumour within the surrounding stroma, blockade of expansive and invasive growth may produce a state like basal-cell carcinomas, which form an enveloping capsule usually staining for reticulin. Such tumours are potentially invasive but, if unable to lyse their capsule, remain benign and undergo maturation and keratinization. By reducing lysis of reticulin-like components in capillary perithelium, enzyme inhibition may contribute to vascular integrity, reduce the incidence of haemorrhage round tumours, and reduce the number of malignant cells penetrating the circulatory system, as reported for ICRF-159 in a metastasizing mouse tumour by Salsbury *et al.* (1970). A synergistic combination of the enzyme-inhibitors ICRF-159 and o-phenanthroline markedly protected tumour-bearing mice from metastasization (Boggust, 1976).

Taken in conjunction with the cytostatic and cytotoxic effect of these drugs on the proliferating cells of the tumour and its stroma, it is possible that observed remissions in tumour viability may be due to the interaction of several factors including those outlined above.

Financial support from the Medical Research Council of Ireland and Saint Luke's Cancer Research Fund is gratefully acknowledged. Appreciation is expressed to Professor M. J. O'Halloran, Medical Director, Saint Luke's Hospital, Dublin and to Dr R. Carroll, Pathologist, who made tissues available for study. Chemically pure thiotepa (Lederle) was obtained by courtesy of Messrs T. P. Whelehan, Son & Co. Ltd.

### REFERENCES

Birbeck, M. S. C. & Wheatley, D. N. (1965) An electron microscope study of the invasion of ascites tumour cells into the abdominal wall. *Cancer Res.*, **25**, 490.

Boggust, W. A. (1976) Factors related to tumour spread in the body. In *6th. Int. Symp. Biol. Characterisation of Human Tumours.* Eds W. Davis and C. Maltoni. Amsterdam: Excerpta Medica. p. 383.

Cameron, E. (1966) *Hyaluronidase and Cancer.* Oxford: Pergamon Press.

Carter, R. L. (1976) Metastasis. In *Scientific Foundations of Oncology.* Eds. T. Symington and R. L. Carter. Ch. 21. London: Heinemann. p. 172.

Creighton, A. M., Hellman, K. & Whitecross, S. (1969) Antitumour activity in a series of *bis*-Diketopiperazines. *Nature*, **222**, 384.

*Documenta Geigy Scientific Tables, 6th Ed.* (1962) Ed. K. Diem. Manchester: Geigy Pharmaceutical Co. Ltd. p. 314.

Easty, G. C. & Easty, D. M. (1976) Mechanisms of tumour invasion. In *Scientific Foundations of Oncology.* Ch. 20. Eds T. Symington and R. L. Carter. London: Heinemann. p. 167.

Frithiof, L. (1972) Ultrastructural changes at the epithelial-stromal junction in human oral pre-invasive and invasive carcinoma. In *Tissue Interactions in Carcinogenesis.* Ed. D. Tarin. London: Academic Press. p. 161.

Goetz, I. E., Weinstein, C. & Roberts, E. (1972) Effects of protease inhibitors on growth of hamster tumor cells in culture. *Cancer Res.*, **32**, 2469.

Hamperl, H. (1967) Early invasive growth as seen in uterine cancer and the role of the basal membrane. In *Mechanisms of Invasion in Cancer*, U.I.C.C. Monographs, Vol. 6. Ed. P. Denoix. Berlin: Springer-Verlag. p. 17.

Keiditsch, E. & Strauch, L. Collagenase activity in certain tumours. *F.E. Biochem Soc.* (Prague). Abstract 75.

Keiditsch, E. & Strauch, L. (1970) Peptidase and collagenase activities in the invasion zones of tumours of the breast. In *Chemistry and Molecular Biology of the Intercellular Matrix, Vol.* 3. Ed. E. A. Balaz. London: Academic Press. p. 1671.

Langer, E., Keiditsch, E., Strauch, L. & Hannig, K. (1968) The activity of collagenase in the simple solid carcinoma of the breast. *Verh. Dtsch. Ges. Pathol.*, **52**, 438.

Latner, A. L., Longstaff, E. & Turner, G. A. (1974) Anti-tumour activity of aprotinin. *Br. J. Cancer*, **30**, 60.

McCroskery, P. A., Richards, J. F. & Harris, E. D. (1975) Purification and characterization of a collagenase extracted from rabbit tumours. *Biochem. J.*, **152**, 131.

*Martindale, The Extra Pharmacopoeia, 26th Ed.* (1972) Ed. N. W. Blacow. London: Pharmaceutical Press. pp. 1193, 819, 403.

Nagai, Y., Sakakibara, S., Noda, H. & Akabori, S. (1960) Hydrolysis of synthetic peptides by collagenase. *Biochim. Biophys. Acta*, **37**, 567.

Nagai, Y., Lapiere, C. M. & Gross, J. (1966) Tadpole collagenase. Preparation and purification. *Biochemistry*, **5**, 3123.

Pearse, A. G. E., Hess, R. & Snyder, R. (1961) Stromal and tumour cathepsins and their relationship to metabolic activity and invasiveness. In *Biological Interactions in Normal and Neoplastic Growth.* Henry Ford Hospital Symposium. Eds. M. J. Brennan and W. L. Simpson. London: Churchill, p. 657.

Peters, L. J. (1975) The natural history of metastasis of a syngeneic murine squamous carcinoma and the prognostic implications of primary tumour size and duration of growth. *Br. J. Cancer*, **32**, 366.

Rassegna Medical and Cultural Review (1971) Ed. G. Gordoni. Milan: Panorama. p. 3, **48**, 4.

Robertson, D. M. & Williams, D. C. (1969) *In vitro* evidence of neutral collagenase activity in an invasive mammalian tumour. *Nature*, **221**, 259.

Salsbury, A. J., Burrage, K. & Hellmann, K. (1970) Inhibition of metastatic spread by ICRF-159; selective deletion of a malignant characteristic. *Br. Med. J.* IV, 344.

Seifter, S. & Gallop, P. M. (1962) Collagenase from Clostridium histolyticum. In *Methods in Enzymology, Vol. 5.* Ed. S. P. Colowick and N. O. Kaplan. New York: Academic Press. p. 659.

Strauch, L. (1967) Nouveaux aspects dans le domaine des enzymes collagenolytiques. *Arch. Biochem. Cosmetol.*, **10**, 11.

Strauch, L. (1972) The role of collagenases in tumour invasion. In *Tissue Interactions in Carcinogenesis.* Ed. D. Tarin. London: Academic Press. p. 399.

Strauch, L. & Vencelj, H. (1967) Collagenase in mammalian cells. *Z. Physiol. Chem.*, **348**, 465.

Strauch, L., Vencelj, H. & Hannig, K. (1968)

Kollagenase in Zellen hoher Entwickelter Tiere. *Z. Physiol. Chem.*, **349**, 171.

SYLVEN, B. (1961) The host-tumour interzone and tumour invasion. In *Biological Interactions in Normal and Neoplastic Growth.* Henry Ford Symposium. Eds. M. J. Brennan and W. L. Simpson. London: Churchill. p. 635.

WASSERMAN, T. H., FRIEDMAN, M. A., SLAVIK, M. & CARTER, S. K. (1973) (±) 1,2-di(3,5-dioxopiperazin-1-yl) propane ICRF-159. *Nat. Cancer Inst. Monogr.*, Clinical Brochure NSC-129943.

WERB, Z., BURLEIGH, M. C., BARRETT, A. J. & STARKEY, P. M. (1974) Inhibition of mammalian collagenases and other metal proteinases. *Biochem. J.*, **139**, 359.

WUNSCH, E. & HEIDRICH, H. G. (1963) Zur Quantitativen Bestimmung der Kollagenase. *Z. Physiol. Chem.*, **333**, 149.